# Astrocytes in Multiple Sclerosis—Essential Constituents with Diverse Multifaceted Functions

**DOI:** 10.3390/ijms22115904

**Published:** 2021-05-31

**Authors:** Rina Aharoni, Raya Eilam, Ruth Arnon

**Affiliations:** Department of Immunology, The Weizmann Institute of Science, Rehovot 761001, Israel; raya.eilam@weizmann.ac.il (R.E.); ruth.arnon@wiezmann.ac.il (R.A.)

**Keywords:** astrocytes, multiple sclerosis (MS), experimental autoimmune encephalomyelitis (EAE), inflammation, astrocyte activation, blood–brain barrier (BBB), tissue damage, neurotrophic factors, neuroprotection, repair processes

## Abstract

In multiple sclerosis (MS), astrocytes respond to the inflammatory stimulation with an early robust process of morphological, transcriptional, biochemical, and functional remodeling. Recent studies utilizing novel technologies in samples from MS patients, and in an animal model of MS, experimental autoimmune encephalomyelitis (EAE), exposed the detrimental and the beneficial, in part contradictory, functions of this heterogeneous cell population. In this review, we summarize the various roles of astrocytes in recruiting immune cells to lesion sites, engendering the inflammatory loop, and inflicting tissue damage. The roles of astrocytes in suppressing excessive inflammation and promoting neuroprotection and repair processes is also discussed. The pivotal roles played by astrocytes make them an attractive therapeutic target. Improved understanding of astrocyte function and diversity, and the mechanisms by which they are regulated may lead to the development of novel approaches to selectively block astrocytic detrimental responses and/or enhance their protective properties.

## 1. Introduction

### 1.1. Multiple Sclerosis and Its Animal Model—Experimental Autoimmune Encephalomyelitis

Multiple sclerosis (MS) is a chronic disease of the central nervous system (CNS), and one of the most prevalent neurological disorders leading to chronic disability among young adults [1]. Patients show a variety of physical and cognitive disabilities, as well as different disease progressions. The most typical progression pattern is an initial phase of relapsing and remitting symptoms (relapsing–remitting MS, RRMS) that can develop to a progressive disease (secondary progressive MS, SPMS). A fraction of the patients show a progressive course from the disease onset (primary progressive MS, PPMS), which represents a somewhat different pathology [2]. The definite etiology of MS is still unknown, but it is established that complex interactions between environmental factors and multiple genes, such as the major histocompatibility complex (MHC) class II HLA-DRB1 gene, HLADRB1* 15:01, are involved [1].

MS is considered an autoimmune disease in which the immune system reacts against the body’s own constituents, in this case against the myelin envelope that surrounds the axons, initiating a vicious inflammatory cascade and formation of the characteristic lesions [1,3,4]. The immunological attack is mediated by autoreactive effector T-cells, T-helper (Th)-1 and Th-17, that recognize specific myelin antigens. Immune cells of both the adaptive and innate immune systems, including cytotoxic T-cells, B-cells, and monocytes/macrophages, are also involved in the inflammatory network and inflict myelin damage [1,3,4]. In addition to the inflammation and demyelination, axonal and neuronal pathologies are crucial components of this disease, in particular during the progressive stage in which degeneration prevails [2,4]. The inflammatory process is modulated by the anti-inflammatory arm of the immune system, T-cells of the Th-2 pathway, regulatory T-cells (Tregs), and regulatory B-cells (Bregs) [3,4,5,6]. In an attempt to overcome myelin damage and degeneration, repair processes such as remyelination and neuroprotection are stimulated [4,7]. Therapeutic approaches aim to suppress the detrimental inflammation and enhance counteracting anti-inflammatory and neuroprotective repair processes in the CNS [1,3,4].

Essential data on MS have been obtained using an animal model, experimental autoimmune encephalomyelitis (EAE), induced by injection of myelin antigens such as myelin basic protein (MBP), myelin oligodendrocyte glycoprotein (MOG), or myelin proteolipid protein (PLP), emulsified in enriched adjuvant, accompanied by administration of pertussis toxin [4,8]. This leads to the priming of myelin-specific T-cells in peripheral lymphoid organs, their differentiation into effector T-cells (mostly Th1 and Th17) and their entry into the CNS, where they are reactivated via interaction with specific myelin antigens on local antigen-presenting cells (APCs). These events are followed by secretion of pro-inflammatory mediators from T-cells, microglia, and astrocytes, which further amplify the inflammatory response, recruit additional immune cells and inflict demyelination and tissue damage.

The encephalitogenic epitopes of the EAE-inducing myelin antigens were identified. Consequently, the disease can be induced by injecting the corresponding peptides. Notably, EAE induction by different encephalitogenic peptides in susceptible animal strains leads to the development of different disease forms that resemble the different MS phases. For instance, the characteristic clinical symptoms manifested as ascending paralysis (from tail to limp), occur in a chronic manner in C57BL/6 mice injected by the peptide corresponding to amino acids 35–55 of MOG, and in a relapsing–remitting manner in SJL/J mice injected by amino acids 139–151 of PLP [9]. An immune response to these peptides occurs in the blood and cerebrospinal fluid of MS patients [10], suggesting that these epitopes are also involved in the human disease. Although the extent of the similarity between EAE and MS is debated, it is accepted that they share essential pathological features, such as inflammation, demyelination, and neuro/axonal damage, as well as activation of microglia and astrocytes [4,8,9]. Consequently, the various EAE models have been highly valuable for studying the pathological mechanisms involved in MS, as well as for drug development.

### 1.2. Astrocytes in CNS Homeostasis

Astrocytes (astroglia), the most abundant cell type in the CNS, were simplistically regarded for many decades as a homogeneous cell population that support the neurons. However, in-depth study revealed that the morphology of astrocytes is complex and region-dependent, implying heterogeneity in structure, distribution, and function of this population [11,12]. Over the last two decades, knowledge of astrocytes has been continuously expanding revealing their fundamental roles in CNS development and function. It is currently clear that astrocytes are versatile dynamic cells, expressing numerous receptors, which enables them to respond to neuroactive compounds, such as neurotransmitters, neuropeptides, growth factors, cytokines, and toxins [13]. With their highly ramified processes that contact the neurons’ pre- and post-synaptic terminals, astrocytes regulate synaptic formation, activity, and plasticity [14]. Astrocytes provide energy substrates and trophic factors for neurons and oligodendrocytes, and maintain the extracellular milieu composition, pH, and electrolyte balance via specialized water and ion channels [13,15]. They also control the neuronal microenvironment by secreting or removing active factors, which trigger receptors on neurons, glia, and blood vessels [13]. These feed-back and feedforward signaling effects fine-tune the balance of neurons between excitation and inhibition [16]. Lipid metabolism is also regulated by astrocytes, which are the primary source of CNS cholesterol, needed for membrane and myelin synthesis [17].

Astrocytes are key components for the functionality of the blood brain barrier (BBB), which is constructed by endothelial cells [ECs], their well-developed tight junctions (TJs), pericytes, and basement membrane [18]. These elements jointly preserve the selective transport properties vital for brain homeostasis, constituting the first line of defense against leukocyte invasion from the periphery. Astrocytes also constitute the glia-limiting membrane (glia limitans) of the meningeal brain barriers, a dense meshwork of astrocytic processes covered by basal lamina that contact the pia mater, and regulate the entry of molecules and cells into the brain parenchyma [18]. Interacting with endothelial cells, pericytes, and neighboring neurons, astrocytes form the neurovascular unit (NVU) that controls the BBB permeability [19]. Neural functionality and survival depend on the delivery of supplements such as oxygen, ions, and glucose through the blood supply, and their adjustment according to the neural activity. By extending and wrapping their endfeet around the cerebral vasculature, perivascular astrocytes exchange glucose, ions, and soluble factors with the endothelial cells, regulating neuronal metabolism [20,21]. Several studies indicate that astrocytic branches can sense neuronal synaptic activity levels, integrate this information, and transmit it to adjacent blood vessels [22,23]. The neuro–hemodynamic coupling role of astrocytes is further supported by our finding that astrocytes, rather than blood vessels, show a striking morphological homology to the neuronal functional boundaries [24]. The anatomical confinement of the astrocytic processes to the neurons was found in multiple auditory and visual systems across different mammalian species—mouse, rat, and human—in an arrangement that enables blood-flow adjustments to the neuronal activity.

In parallel to the advance in understanding astrocytes’ contribution to brain function and homeostasis, their activity under pathological circumstances, particularly in MS, was investigated. Consequently, their view as secondary bystanders, that respond to insult by forming a glial scar, but play little role in disease mechanism, dramatically changed [25]. Studies, utilizing novel technologies in MS patient samples and in the EAE model, have revealed the detrimental and the protective roles of this multifaceted cell population in facilitating inflammation, tissue damage, and repair. In the following, we discuss several significant aspects of astrocyte involvement in the pathological as well as in the modulatory and repair processes occurring in MS/EAE.

## 2. Astrocyte Activation by Inflammatory Stimulation

The substantial morphological changes which astrocytes undergo under various pathological conditions were noted more than a century ago [26]. Astrocytes respond to CNS injury and disease by a complex process of activation that integrates morphological changes (cell body and process hypertrophy) and transcriptional and biochemical transformations, as well as robust functional modifications, accompanied by a reduction in homeostatic metabolic functions. This is associated by upregulated expression of the intermediate filament glial fibrillary acidic protein (GFAP), which is therefore used as a marker of reactive astrocytes (astrogliosis).

**Figure 1:**
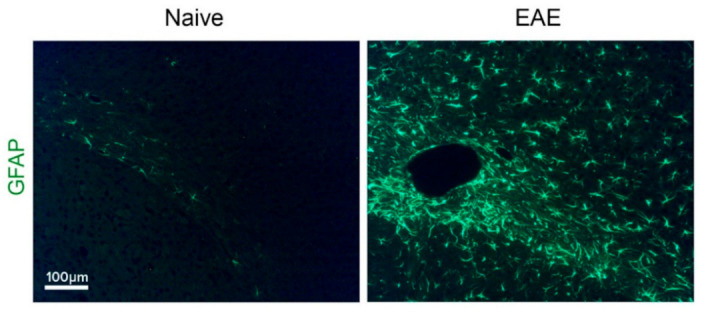
Astrogliosis in the somatosensory cortex of an EAE-induced mouse in comparison to a naïve mouse. Immunohistochemical staining for GFAP, 15 days after EAE-induction.

In MS and EAE, astrocyte activation is a widespread characteristic (Figure 1), initiated at a very early stage of the disease, persisting into the chronic phase, and continuing even after the decline of immune cell presence [25,27]. Detection of activated astrocytes in the frontal cortex at an early EAE stage, before leukocyte infiltration, is attributed to spreading of inflammatory factors across the BBB [28,29]. Thus, T-cells primed in peripheral lymphatic organs reach the CNS borders, undergo re-stimulation by perivascular/meningeal APCs, and produce pro-inflammatory cytokines, such as tumor necrosis factor (TNF), interleukin (IL)-17, granulocyte–macrophage colony-stimulating factor (GM-CSF), and interferon (IFN)-γ, that activate adjacent CNS-resident cells, particularly astrocytes and microglia. Cytokines, chemokines, and growth factors, secreted by leukocytes and local CNS cells orchestrate the cascade of molecular, cellular, and functional changes referred to as astrocyte activation [30]. Under the inflammatory demyelinating conditions in MS/EAE lesions, astrocytes proliferate and form glial scars composed of a dense network of hypertrophic cells, expressing high levels of adhesion molecules, cytokines, growth factors, receptors, enzymes, and protease inhibitors that modify the surrounding extracellular matrix (ECM) [31].

**Figure 2:**
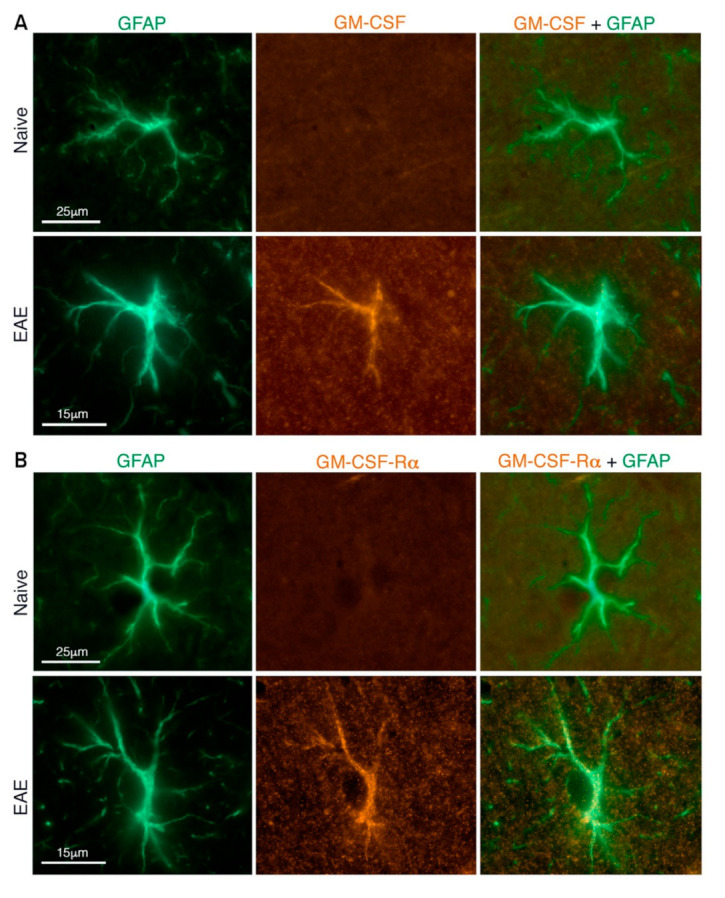
Astrocyte expression of GM-CSF and its receptor (subunit α) GM-CSFRα. Representative immunohistochemical images from the somatosensory cortex (layer 4). (**A**) GM-CSF expression and (**B**) GM-CSFRα expression by GFAP expressing astrocytes in EAE-affected mice, but not in naïve mice. Reprinted with permission from Eilam et al. (2018). Copyright Year 2021, John Wiley and Sons.

Activated astrocytes produce or upregulate the secretion of various factors. These include cytokines e.g., TNF-α, IL-1β, IL-6, and GM-CSF (Figure 2A); chemokines e.g., CCL2, CCL3, CCL5, CCL20, CXCL10, and CXCL12; and neurotrophic growth factors e.g., nerve growth factor (NGF), brain-derived neurotrophic factor (BDNF), vascular endothelial growth factor (VEGF), and leukemia inhibitory factor (LIF) [30,31,32,33]. Upregulated expression of cytokine receptors such as the GM-CSF receptor in reactive astrocytes further augment their response to the cytokines secreted by immune cells in their surroundings [34] (Figure 2B). Reactive astrocytes also express cell adhesion molecules such as ICAM-1 and VCAM-1 [33,35], inducible nitric oxide synthase (iNOS) with concomitant production of reactive nitrogen species [36,37], and toll-like receptor 3 (TLR3) [38].

In accordance, upregulation of multiple genes linked to innate and adaptive immunity, antigen presentation, leucocyte attraction and invasion (particularly chemokines and cytokines), was found in astrocytes upon in vitro inflammatory stimulation [39]. Mitochondrial dysfunction, in particular metabolic shift from oxidative phosphorylation to glycolysis, is associated with inflammatory activation of astrocytes [40]. Using novel genomic technologies for characterization of astrocyte heterogeneity, it has been recently demonstrated that specific astrocyte subsets or their activation states are associated with specific genomic programs and functions [41].

Several signaling-regulation pathways are triggered in activated astrocytes [42,43,44], particularly, the canonical nuclear factor-κB (NF-κB) pathway, which is essential for driving the inflammation in CNS diseases. EAE is ameliorated by astrocyte specific ablation of key signaling molecules related to the NF-κB signaling cascade [45,46,47]. Moreover, a genetic variant with enhanced NF-κB activation in astrocytes is associated with MS [48]. Additional functional regulators of astrocyte reactivity are signal transducer activator of transcription 3 (STAT3) [49,50] and astrocytic nuclear factor 1A (NF1A) [51]. It is still unclear what molecular cues drive the expression of these transcription factors, how they interact, and if they underlie the formation of unique types of reactive astrocytes.

It should be noted that the above-described spectrum of changes is context-specific that they vary depending on the nature and severity of the CNS insults [31,52,53]. Thus, reactive astrocytes with the potential to release diverse potent molecules and impact nearby cells, can exert pro-inflammatory effects and aggravate the neurological impairment, or exert anti-inflammatory effects, promoting protection and repair. Accordingly, reactive astrocytes have been categorized as “A1” or “A2” [54], in an analogy to the “M1/M2” categories adopted for macrophages and microglia. A1-type astrocytes, induced by inflammation, are toxic to neurons and oligodendrocytes [54,55]. In contrast, A2-type astrocytes have been attributed anti-inflammatory and neuroprotective properties [54,56]. This concept, however, is oversimplified, as reactive astrocytes exceed this dichotomy and adopt a range of profiles [57,58]. Furthermore, as described in the following, reactive astrocytes display multiple opposing functions, indicative of their multifaceted heterogeneity. Contemporary studies using advanced genomic characterization exposed different astrocytic subsets displaying distinct functions, with a certain plasticity between them [58,59]. This heterogeneity is shaped by interactions with other cell types in the inflamed CNS [41,58].

## 3. Astrocytes as Mediators of MS and EAE Pathology

Astrocytes on the CNS borders, as well as in the lesion sites where they comprise the most prevalent cell type, play detrimental roles at various critical points of MS/EAE pathogenesis, constructing inflammatory cascades and inflicting tissue injury. At the lesion margins, reactive astrocytes expressing multiple pro-inflammatory and neurotoxic mediators extend into adjacent normal-appearing matter, indicative of their participation in lesion expansion.

### 3.1. Constructing the Inflammatory Cascade

Reactive astrocytes play fundamental roles in recruiting, instructing, and retaining leukocytes at the lesion sites, engendering the positive-feedback inflammatory loop that mediates the disease [18,27,30]. Astrocyte activation occurs early during lesion development, before significant numbers of inflammatory cells enter the CNS [25,27,28], suggesting that astrocytes facilitate the large-scale influx of leukocytes. Infiltration of leukocytes into the CNS parenchyma is not a passive process, but requires active recruitment across specialized barriers. Astrocytes are uniquely positioned to regulate this recruitment, as they regulate the BBB permeability and form the glia limitans [18,20,60]. BBB dysfunction or breakdown is a characteristic feature of MS (in particular of the relapsing–remitting stage) as well as of EAE [61]. This is manifested by a reduction in the tight junction proteins, claudins and occludin, with subsequent increases in BBB permeability [60,62]. Cytokines, chemokines, matrix metalloproteinases (MMPs), and reactive oxygen species (ROS) augment BBB permeability [61,63]. Reactive astrocytes release factors such as IL-1β, TNF, glutamate, and nitric oxide (NO) that downregulate protein expression on the tight junction surface and promote endothelial cells apoptosis [64,65,66]. Astrocytes are also a major source of CC–chemokine ligand 2 (CCL2), which induces internalization of the tight junction proteins, resulting in reduced surface levels of claudin-5 and occludin [64,67,68]. In addition, astrocyte-derived vascular endothelial growth factor A (VEGF-A) and thymidine phosphorylase (TYMP) are key permeability factors that promote BBB breakdown by repressing tight junction protein expression [69]. In parallel, a reduction in the “protective factor” angiotensinogen (AGT), required for intact localization and function of occludin, has been detected in astrocytes activated in vitro by pro-inflammatory cytokines, as well as in perivascular astrocytes within MS lesions [70].

**Figure 3:**
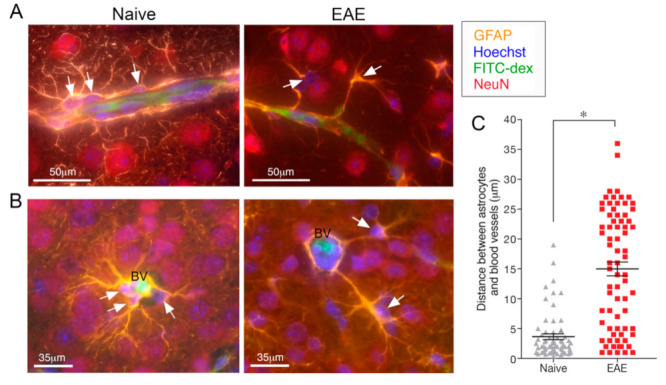
Reactive astrocytes detach from the perivascular vicinity and lose the endfeet coverage around the blood vessels (BV). Immunohistochemical depiction of the neurovascular unit in the motor and somatosensory cortex (layer IV). The lumen of the microvessels is visualized by its FITC-dextran content, astrocytes by GFAP expression, neurons by NeuN expression, and overall cell nuclei by Hoechst staining. Representative images from naïve and EAE mice, 21 days after EAE induction. (**A**) Longitudinal sections along blood vessels and (**B**) cross sections of penetrating blood vessels. Arrows indicate location of astroglial cell bodies. (**C**) Quantification of the distances between individual astrocytic cell bodies and their neighboring blood vessels. * *p* < 0.05. Adapted with permission from ref. [71]. Glia 2018, 66, 1098-1117. Copyright Year 2021, John Wiley and Sons.

The structural architecture of the neurovascular unit controlling the BBB permeability [19], and the glia limitans formed by the astrocytic endfeed [18], is disrupted upon astrocyte activation. We have demonstrated that unlike in naïve mice, in which most of the microvasculature surface is ensheathed by ramified endfeet, in EAE-activated perivascular astrocytes retract their processes and cease their endfeet coverage around blood vessels [71] (Figure 3). Furthermore, cross-sectional analysis of penetrating vessels indicated that in EAE, in addition to the retraction of the astrocyte processes, the astroglial cell bodies detach from the perivascular vicinity [71] (Figure 3). Leukocytes in the perivascular spaces contribute to this detachment by producing MMPs, which, cleave dystroglycan, the anchor between the endfeet of astrocytes and the basement membrane [72]. Reduced GFAP immunoreactivity and damage to the astrocytic endfeet vascular coverage have also been reported in MS patients [25,73,74]. These combined processes indicate that loss of astrocyte function at the vascular interface is a component of EAE/MS disease mechanism, resulting in BBB breakage and massive infiltration of immune cells into the CNS.

In addition to their effect on BBB permeability, astrocytes also “actively” recruit leukocytes into the CNS parenchyma by producing chemoattractant molecules and inducing an increased expression of adhesion molecules on the endothelial cells. As described in Section 2, astrocytes respond to inflammatory stimulation by producing various chemokines such as CCL2, CCL3, CCL5, CCL20, CXCL10, and CXCL12 [31,32,33]. Astrocyte-derived CCL2, CCL20, and CXCL10 were shown to play important roles in the recruitment and accumulation of immune cells into the CNS during EAE [75,76,77]. Increased astrocytic CXCL12 expression in MS tissue specimens is associated with CNS inflammation [78]. Furthermore, IL-6 and tissue plasminogen activators (tPA), produced by reactive astrocytes, upregulate adhesion molecules on the endothelial cells, such as E-selectin, VCAM, ICAM, and CD44 ligand hyaluronan, resulting in increased endothelial–T-cell binding [79,80]. Decreased expression of connexin 43 (Cx43) in activated astrocytes is also linked to elevated adhesion molecule expression in the endothelial cell [81], and to rapidly progressive MS [82].

An additional manner by which astrocytes exacerbate the inflammation is by facilitating restimulation of the infiltrating immune cells in situ, acting as a local component of the innate immune system. The capability of astrocytes to act as antigen-presenting cells and restimulate effector T-cell is controversial. Immunohistochemical staining revealed that a subset of astrocytes in active MS lesions expresses MHC class II molecules, supporting potential antigen presentation [83]. Yet, in another study, astrocytic MHC class I, but not class II expression, was found in active MS lesions, suggesting antigen presentation to class I-restricted cytotoxic T-cells, but not to CD4+ T-cells [84]. In vitro studies in murine-activated astrocytes indicate that they are able to process and present myelin antigens such as MBP to T-cells [85]. Interestingly, astrocytes efficiently presented the immunodominant epitope PLP139-151, but not subdominant PLP peptides [86]. In the case of MOG, astrocytes presented both the immunodominant and subdominant peptides, but not the native MOG protein [87]. Likewise, there are conflicting reports regarding the expression of costimulatory molecules on astrocytes [88,89,90], so their actual function as fully competent APCs is not yet clear. Regardless, astrocytes can enhance polarization of CD4+ T-cells to Th1/Th17 phenotypes, thus enhancing their pro-inflammatory functions [91,92,93]. They can also produce IL-15, resulting in activation of cytotoxic CD8+ T-cells that exacerbate tissue damage [94]. Furthermore, factors such as IL-15 and the B-cell-activating factor BAFF produced by astrocytes, support proliferation, differentiation and survival of B-cells, which are currently recognized as important contributors to MS pathology [95,96,97]. In addition to their effect on immune cells, factors such as IL-6, and lymphotoxin-alpha (LT-α) produced by astrocytes, promote activation and accumulation of microglia [98,99]. These combined effects of reactive astrocytes generate an inflammatory-promoting environment in the MS lesions that sustains and exacerbates the pathological process. Notably, astrocytes comprise one component in the complex multilayered crosstalk between penetrating immune cells and CNS-resident cells that stimulate each other by releasing inflammatory substances. For example, GM-CSF secreted by immune cells in the MS lesions can efficiently affect microglia and astrocytes, which in parallel upregulate GM-CSF receptor expression [34].

### 3.2. Inflicting Tissue Damage

Astrocytes in the inflamed CNS, along with leukocytes and microglia, create a hostile environment, rich in pro-inflammatory and toxic factors that inflict damage to myelin, oligodendrocytes, and neurons, by various routes. Reactive oxygen species (ROS) and reactive nitrogen species (RNS) induce, when in excess, oxidative stress, mitochondrial injury, and impaired energy metabolism [37,100,101,102]. Astrocytes are major producers of ROS and RNS, and this production is triggered during CNS inflammation [30,37,41,103]. Pro-inflammatory cytokines, such as IL-1β, IL-17, and IFN-γ induce the expression of the NO-generating enzyme iNOS in astrocytes, leading to additional NO accumulation [36,104,105]. Furthermore, elevated levels of BDNF and upregulation of its receptor, tropomyosin-related kinase B (TrkB) on activated astrocytes results in excessive NO production and NO-driven neurotoxicity [106]. Reactive astrocytes also promote oligodendrocyte and neuronal death through defective control of neurotransmitter uptake and release. Under normal conditions, astrocytes regulate glutamatergic neurotransmission by eliminating glutamate from the CNS extracellular milieu. In reactive astrocytes, the expressions of glutamate transporters and enzymes responsible for glutamate degradation are reduced, resulting in excessive extracellular glutamate and excitotoxic injury [107,108].

**Figure 4:**
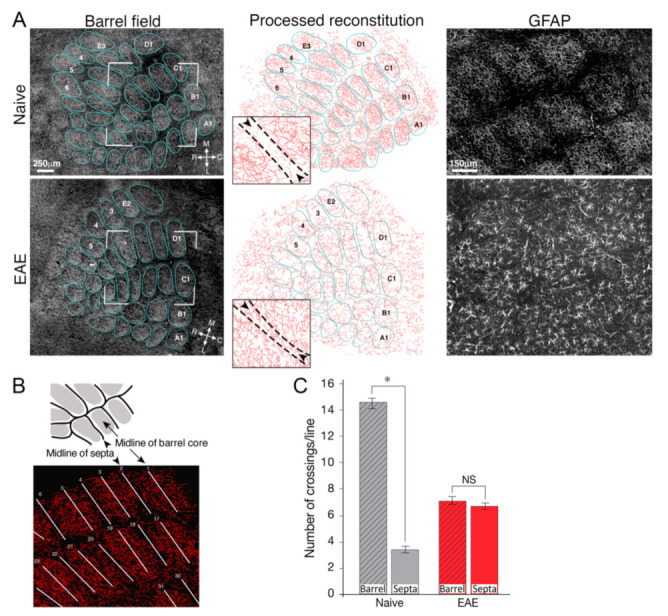
Loss of astrocytic confinement to the neuronal boundaries under inflammation. Representative images from layer IV of naïve and EAE mice, 21 days after EAE induction. (**A**) Left column: dark field illumination of the barrel field area. Middle column: reconstruction of the astrocyte processes performed by manually drawing their branches from GFAP-stained astrocytes. Clear gaps in astrocyte distribution at the barrel boundaries are visible in naïve, but not in EAE mice. Right column: images of astrocytes at high magnification stained with GFAP. (**B**) Schematic drawing of the barrel field, and an example of lines drawn at the septa centers or the barrel cores. (**C**) Quantitative analysis of astrocyte processes crossing the line drawing at the septal and barrel cores. * *p* < 0.001; NS, not significant. Adapted with permission from ref. [71]. Glia 2018, 66, 1098–1117. Copyright Year 2021, John Wiley and Sons.

Devoid of myelin protection and support, axons and neurons in the demyelinated lesions are particularly prone to injury inflicted by the hostile inflammatory milieu. Besides reactive species secretion and excitotoxic injury, astrocytes produce a variety of harmful mediators such as proteolytic enzymes, cytokines, and complement components [33,94,109]. Furthermore, under inflammatory conditions, the connection between astrocytes and neurons is impaired and astrocytes fail in their essential role of neuro–hemodynamic coupling. We examined individual astrocytic processes in layer IV of the somatosensory cortex, where the well-defined neuronal columns (barrels) are linked to functional properties. In contrast to naïve mice, in which the highly modulated patches of astrocyte processes overlap the barrel cores, in EAE, loss of astrocytic confinement to the neuronal boundaries was evident [71] (Figure 4). Loss of coupling between lactate supplied by the astrocytes and the neurons’ energetic needs, further promotes neurodegeneration [40]. Decreased astrocyte-derived trophic support also contributes to neuronal damage [110]. Finally, the ECM in the glial scar is inhibitory to repair processes such as axon sprouting and synaptogenesis [111,112].

The myelin-producing cells, the oligodendrocytes, are terminally differentiated cells with a limited capacity to respond to injury and survive the toxic conditions within the lesion. Terminally differentiated oligodendrocytes in the demyelinating lesions undergo apoptotic cell death [113]. Accordingly, remyelination requires proliferation of oligodendrocyte progenitor cells (OPCs), their migration into the demyelination sites, and their differentiation into mature myelin-producing oligodendrocytes [114]. In the early stages of MS, OPCs recruitment to demyelination sites often leads to remyelination. However, at later disease stages, in particular in chronic lesions, remyelination is impaired and limited to a small rim at the lesion border [113]. Astrocytes contribute to the failure of remyelination in several ways: The glial scar formed by reactive astrocytes poses a physical barrier that blocks OPCs migration into the demyelinated area, so OPCs are practically sequestered at the lesions edge [115]. The glial scar also generates a biochemical obstacle, as astrocytes secrete different substances that interfere with remyelination. For instance, hyaluronan produced by astrocytes, accumulates in chronic MS and EAE demyelinated lesions and inhibits OPC maturation [116]. Chondroitin sulfate proteoglycans (CSPG), produced by reactive astrocytes at the border of demyelinating areas, inhibits OPC process outgrowth, differentiation, and adhesion [117]. Fibronectin, an ECM glycoprotein, secreted by astrocytes in chronic MS lesions, hinders oligodendrocyte differentiation and remyelination [118]. Additional factors released by reactive astrocytes that were shown to interfere with OPCs differentiation are fibroblast growth factor-2 (FGF-2) [119] and cytokines, such as TNF-α, and IL-6 [120,121]. Moreover, reactive astrocytes promote apoptosis of oligodendrocytes and OPCs via TNF [122,123] and the Fas ligand [124]. Reactive astrocytes are also a source of proteolytic enzymes, such as calcium-activated neutral protease (calpain), that participate in the degradation of myelin proteins, resulting in destabilization of the myelin sheath [125]. In addition, astrocytes in the spinal cord and optic nerve of EAE mice have been shown to produce fewer cholesterols, which are required for myelin production [17], thereby inhibiting remyelination [126].

## 4. Astrocytes as Mediators of MS and EAE Modulation and Repair

The contribution of astrocytes to protection and repair has been recognized following the development of animal models in which reactive astrocytes could be selectively ablated. In the EAE system, targeted ablation of reactive astrocytes was associated with increased leukocyte infiltration into the CNS parenchyma, and a severe and rapid fulminant clinical course [127]. Indeed, reactive astrocytes employ a variety of strategies to counteract inflammation, limit tissue damage, and support repair processes, thus contributing to disease arrest and to recovery of CNS functions [128,129]. Furthermore, in response to CNS insults, astrocytes receive and carry information to other cells, in a coordinated action to stimulate defense and repair mechanisms [41].

### 4.1. Limiting BBB Damage and Inflammation

Astrocytes have an important role in protecting BBB integrity and restoring its function, restricting leukocytes infiltration into the CNS, and suppressing excessive immune response [18]. In addition to their role in BBB construction (described in Section 1.2), reactive astrocytes are capable of actively affecting the BBB by releasing various factors that reduce its disruption and downregulate immune cell infiltration [128]. This includes production and release of retinoic acid (RA) that induces immune quiescence, attenuates oxidative stress in endothelial cells, and decreases monocyte adhesion [130]. The key enzyme for RA synthesis, retinaldehyde dehydrogenase 2 (RALDH2), is highly expressed in reactive astrocytes in both active and chronic MS lesions [130]. Astrocytes are the main producers of the MMP counter-regulator, tissue inhibitor of metalloproteinase-1 (TIMP-1). The expression of this inhibitor is increased in activated astrocytes around the lesions of EAE-induced mice, counteracting, in situ, the detrimental MMP enzymatic activity [131]. The antioxidant protein peroxiredoxin 6 (PRDX6) that lowers MMP9 expression, fibrinogen leakage, and free radical damage, is also elevated in astrocytes of MS patients and EAE mice [132]. Astrocytes secrete sonic hedgehog (Hh) that, together with the hedgehog receptors expressed by the BBB endothelial cells, decreases the expression of pro-inflammatory mediators as well as the adhesion and migration of leukocytes [133].

The glial scar, formed by a dense network of astrocytes in response to injury, presents a physical barrier that restricts the influx and spread of immune cells from perivascular spaces to the CNS [31,127]. Furthermore, astrocytes maintain the CNS immune privilege by inducing an immunosuppressive environment, and skewing T-cell polarization to a protective Th2 phenotype. Thus, astrocytes, which have a certain potential to function as APCs (discussed in Section 3.1), are more efficient in stimulating Th2 anti-inflammatory responses than Th1 pro-inflammatory responses [134]. Moreover, the anti-inflammatory Th2 hallmark cytokines, IL-10 and IL-4, as well as their receptors, are intensely expressed in reactive astrocytes within and around active and chronic MS lesions [135] as well as in EAE [136]. IL-27, that modulates inflammation, is also expressed by astrocytes in active MS plaques [137]. Astrocytes suppress T-cell activation, proliferation, and effector function by upregulating the inhibitory molecule CTLA-4 on autoreactive T-cells [138]. Upon contact with astrocytes, regulatory anergic T-cells (CD4+ and CD8+) prevent proliferation of mitogen-induced T lymphocytes as well as of autoreactive T-cells [139]. In chronic active MS plaques, reactive astrocytes express the inhibitory membrane glycoprotein CD200, which downregulates immune activity [140]. In addition, ubiquitin-aldehyde-binding 1 (OTUB1), a deubiquitinating enzyme upregulated in astrocytes during MS and EAE, prevents IFN-γ-induced hyperactivation [141]. Notably, at late EAE stages, IFN-γ signaling in astrocytes is neuroprotective, so astrocytes play a role in limiting the inflammation depending on the signaling pathway mediated by the engagement of IFN-γ receptors [142].

Programmed cell death is also a mechanism by which astrocytes eliminate infiltrating inflammatory cells. Thus, apoptotic T-cells colocalize with astrocytes in the CNS parenchyma [143], and galectin-9 in activated astrocytes promotes encephalitogenic T-cell apoptosis [144]. Apoptotic elimination of T-cells from the CNS by astrocytes via Fas ligand (FasL) has been shown to precede EAE recovery [145]. Additional mechanisms by which astrocytes induce inactivation and apoptosis of autoreactive T-cells involve the astrocyte-derived immune suppressor factor (AdIF) [146] and the NO pathway [147]. Furthermore, astrocytes promote the expression and enzymatic activity of CD39 and CD73 ectonucleotidases in activated CD4+ T-cells by a contact-dependent mechanism, resulting in T-cell differentiation to an immunosuppressive phenotype [148].

### 4.2. Detoxification Activities and Prevention of Tissue Damage

In response to stress or toxic conditions inside the lesions, astrocytes constitute a protective barrier that reduces neuronal and oligodendrocytes injury through specialized detoxifying mechanisms. Thus, reactive astrocytes are the main cell type defending against oxidative stress, by enhanced production of an array of antioxidant enzymes in the lesions that decrease ROS-induced cellular damage. These include superoxide dismutase (SOD), catalase, heme oxygenase 1 (HO-1), NAD(P)H:quinone oxidoreductase 1 (NQO1), and peroxiredoxins (PRDXs) [149,150,151]. Transcriptional activation of various antioxidant enzymes is controlled by NF-E2-related factor 2 (Nrf2). The expression of DJ-1, which stabilizes Nrf2 and positively regulates its activity, is increased in astrocytes in MS lesions, enabling antioxidant protection [152]. An additional transcription regulator, proliferator activated receptor gamma coactivator-1alpha (PGC-1α), induced in astrocytes in MS lesions, promotes the expression of mitochondrial antioxidants PRDX3 and Trx2, lowering ROS production [153]. Collectively, these enzymes and transcription factors, expressed by reactive astrocytes in the lesion sites, function as an endogenous protective mechanism that alleviates oxidative damage.

Exposure of astrocytes to mild levels of inflammatory cytokines increases their production of detoxifying cytosolic enzymes and antioxidants, and downregulates detrimental enzymes such as iNOS, thus protecting oligodendrocytes and neurons from NO-induced death [128]. Additional detoxifying activities of astrocytes, shown in models of brain injury, are removal of excessive glutamate from the extracellular space and restoration of ionic homeostasis, as well as protection against glucose-induced metabolic stress, iron toxicity, and DNA damage [129].

### 4.3. Neuroprotection and Repair

For many decades, it was believed that severe astrogliosis and glial scar formation inhibits axonal regrowth and is therefore detrimental for the neurological outcome. However, increasing evidence has revealed that astrocytes also play beneficial roles in supporting and promoting repair processes such as remyelination, axonal regeneration, and neurogenesis. Astrocytes provide critical trophic support for neurons and oligodendrocytes by supplying an enormous array of neurotrophic factors, neuropoetic cytokines, and growth factors, with tissue-protective and regenerative properties [154]. Increased astrocytic production of various such factors has been demonstrated both in MS and EAE [155]. For example, BDNF which maintains neuronal survival and regulates oligodendrocytes generation and remyelination, is found in reactive astrocytes within MS and EAE lesions [156,157,158]. Ablation of BDNF specifically in astrocytes, exacerbates EAE and increases axonal damage, but does not affect immune cell infiltration, confirming the neuroprotective activity of astrocytic-derived BDNF [156,159]. It should be noted that astrocytes, as such, respond to increased levels of BDNF by secreting neurotoxic amounts of NO, indicative of the dual putative protective and degenerative roles of astroglia [106]. The expression of ciliary neurotrophic factor (CNTF), a survival factor for neurons, that supports oligodendrocyte maturation, is increased in astrocytes within white matter lesions of MS patients [160]. CNTF-deficient mice manifested severe EAE with poor recovery, decreased OPC numbers, and increased oligodendrocyte apoptosis compared to wild-type mice [161]. In a virus-induced demyelination model, the increase of CNTF in astrocytes within and around lesions coincided with spinal cord remyelination and recovery [162]. CNTF is an upstream regulator of fibroblast growth factor-2 (FGF2), a potent mitogen of OPCs, and an inducer of neurogenesis [162,163]. In EAE, elevated FGF2 levels in white matter astrocytes were detected at the initial remyelination phase [164]. In a lysolecithin-induced demyelination model, FGF2 enhanced hippocampal myelination as well as the recruitment of OPCs and neural stem cells to the lesion area [165]. IL-11, expressed by reactive astrocytes in MS lesions, has been shown to promote neuronal differentiation, oligodendrocyte occurrence, and myelin formation [166].

The various neurotrophic factors, cytokines, and growth factors, supplied by astrocytes, facilitate the key reparative process in the context of MS/EAE, remyelination, by supporting OPCs proliferation, migration into demyelinated sites, and differentiation to myelin-producing oligodendrocytes [114]. Interestingly, astrocytes as such may constitute a potential backup reservoir for oligodendrocytes [167,168]. Additional support that astrocytes provide for remyelination is the recruitment of macrophages/microglia [32,75,76,77] that remove the myelin debris from the demyelinated lesion sites, enabling the formation of new myelin. Astrocyte ablation that prevents the recruitment of microglia cells to demyelination sites, leads to a delayed removal of myelin debris, reduced OPC proliferation, and impaired remyelination [169]. Furthermore, hypertrophic astrocytes at the leading edge of actively demyelinating MS lesions contain myelin, suggestive of their potential activity in myelin phagocytosis [170]. The essential role of astrocytes in remyelination was further emphasized, using an ethidium-bromide-induced demyelination model, by the failure of OPCs to remyelinate in the absence of astrocytes [171], and increased remyelination following astrocyte transplantation [172].

Astrocytes are also important participants in the regeneration processes occurring in the neuronal population, mainly via their production of diverse neurotrophic and growth factors. In addition to the critical trophic support, astrocyte-derived ECM proteins and MMPs can increase axonal regeneration and functional recovery [54]. Indeed, prevention of astrocytic scar formation reduced axonal regrowth [173]. Furthermore, following injury, astrocyte processes envelope the synapses and release neuromodulators that promote synaptogenesis [128]. It has also been recently established that astrocytes are key regulators of neuronal plasticity, a fundamental property of neuronal circuits, allowing them to adapt to alterations in activation [174].

Astrocytes may also function as support cells, stimulating neurogenesis within the specialized niches, the subventricular zone (SVZ) and the hippocampal dentate gyrus, where neural progenitor cells (NPCs) reside in the adult brain. These undifferentiated multipotent cells can migrate beyond their sites of origin and differentiate into mature neurons and glia. In response to EAE induction, neuronal progenitor cells proliferate and accumulate in the damaged areas [175]. Activated astrocytes express growth factor and signaling molecules that regulate stem cell proliferation differentiation and eventual fate, such as FGF-2 and members of the Jagged/Notch and WNT signaling pathways [176,177,178]. Astrocyte-derived EMC molecules such as chondroitin sulfate dermatan sulfate and proteoglycans, abundantly expressed in the neurogenic niches, play a role in controlling neural stem cell fate, maturation, and survival [179]. However, it should be noted that detrimental effects of FGF-2 and chondroitin sulfate on repair processes were also demonstrated [117,119]. Finally, a subpopulation of astrocytes may function as pluri- or unipotential progenitor cells that give rise to neurons, although to an extent which is still unclear in the adult human CNS [25,180].

## 5. Astrocytes as a Therapeutic Target—Modulation of Reactive Astrocytes by MS Treatments

The pivotal roles played by astrocytes make them an attractive target for MS therapy. None of the currently approved MS treatments specifically target astrocytes, but effects on astrocytes have been demonstrated for several therapies. Dimethyl fumarate (DMF) inhibits pro-inflammatory activation of astrocytes, including NF-κB signaling [181], and activates the transcriptional factor Nrf2 that regulates anti-oxidative responses in astrocytes [182]. Fingolimod, an analog of sphingosine-1-phosphate (S1P), which specifically targets sphingosine-1-phosphate receptors (S1PRs), affects astrocytes by inhibiting NF-κB signaling, reducing pro-inflammatory cytokine expression, and enhancing neurotrophic factors [183]. Laquinimod reduces NF-κB signaling and pro-inflammatory responses of astrocytes [184]. IFN-β induces the transcription of suppressor of cytokine signaling (SOCS)-1 and SOCS-3, which inhibit astrocyte hyperactivation [185]. Glatiramer acetate (GA) induces expression of IL-10 and transforming growth factor (TGF-β) by astrocytes [136], restores perivascular astrocyte connections with both the blood vessels and the neuronal synapses [71], and inhibits TNF-α-induced RANTES release from astrocytes [186].

Ideally, astrocyte-directed treatments should take into account their multi-functionality, and attempt to block detrimental responses while enhancing protective properties. The current notion that astrocytes are heterogeneous with respect to the molecules they express and the functions they exhibit, raises the possibility to manipulate specific astroglial populations. Several novel approaches to affecting astrocytic beneficial properties were recently suggested. Stimulation of metabotropic glutamate receptors (mGluR), in particular mGluR3 and mGluR5, which are upregulated in reactive astrocytes, elicits neuroprotective repair processes such as astrocytic BDNF synthesis [187]. Metabolites of dietary tryptophan, produced by the commensal flora, control TGF-α and VEGF-β production, resulting in the modulation of the astrocytic transcriptional program and CNS inflammation [188]. The detection of a metabolic control mechanism that drives pro-inflammatory astrocyte activities through the mitochondrial antiviral signaling protein (MAVS), may lead to identification of new therapeutic targets [189]. Furthermore, a subset of astrocytes expressing the lysosomal protein LAMP12 and the death receptor ligand TRAIL3, which limits CNS inflammation by inducing T-cell apoptosis, has been recently identified [190]. Interestingly, these astrocytes are maintained by meningeal IFN+ NK cells, in which IFN-γ expression is modulated by the gut microbiome, suggesting a novel mechanism for astrocyte modulation. Selective regulation of the diverse astrocyte activities requires further knowledge of their subset heterogeneity and plasticity, as well as deeper understanding of their activation signaling.

## 6. Conclusions

While the pathological inflammatory process in MS/EAE is primarily initiated by bone-marrow-derived components, it is currently clear that astrocytes play essential roles in recruiting, instructing, and retaining these leukocytes at the lesion sites, engendering the positive-feedback inflammatory loop that mediates the disease. Astrocytes also inflict tissue damage through their intrinsic neurotoxic activities and the activation of other cells, thus promoting neurodegeneration and disease progression. Conversely, the roles of astrocytes in restricting detrimental inflammation and in promoting neuroprotection and repair have also been established. These combined effects emphasize the importance of astrocytes as fundamental constituents of MS pathology and regulation. Yet, the multiple, in part opposing, functions raise the notion that astrocytes are bystander “flexible” participants tuned by various context-specific factors, such as the region in which they reside, the nature and the severity of the CNS insults, the local pro/anti-inflammatory milieu, and the crosstalk with immune and resident cells. The current focus in astrocyte biology research is on the heterogeneity and the characterization of specific astrocytic subsets. The diversity of astrocytes with respect to the molecules they express and the functions they exhibit is widely appreciated, while their different regulation and activation signals, as well as their plasticity and communication with neighboring cells, are intensively investigated. Improved understanding of astrocyte diversity and the mechanisms by which they are regulated may lead to identification of novel targets to selectively manipulate astrocytic response, for the development of effective MS treatments.

## Data Availability

Not applicable.
